# Induced Pluripotent Stem Cells-Derived Mesenchymal Stem Cells Attenuate Cigarette Smoke-Induced Cardiac Remodeling and Dysfunction

**DOI:** 10.3389/fphar.2017.00501

**Published:** 2017-07-28

**Authors:** Yingmin Liang, Xiang Li, Yuelin Zhang, Sze Chun Yeung, Zhe Zhen, Mary S. M. Ip, Hung Fat Tse, Qizhou Lian, Judith C. W. Mak

**Affiliations:** ^1^Department of Medicine, The University of Hong Kong Pok Fu Lam, Hong Kong; ^2^Shenzhen Institute of Research and Innovation, The University of Hong Kong Pok Fu Lam, Hong Kong; ^3^Research Centre of Heart, Brain, Hormone and Healthy Aging, The University of Hong Kong Pok Fu Lam, Hong Kong; ^4^Department of Ophthalmology, The University of Hong Kong Pok Fu Lam, Hong Kong; ^5^Department of Pharmacology and Pharmacy, The University of Hong Kong Pok Fu Lam, Hong Kong

**Keywords:** cigarette smoke, induced pluripotent stem cells, inflammation, lipids metabolism, mesenchymal stem cells, oxidative stress

## Abstract

The strong relationship between cigarette smoking and cardiovascular disease (CVD) has been well-documented, but the mechanisms by which smoking increases CVD risk appear to be multifactorial and incompletely understood. Mesenchymal stem cells (MSCs) are regarded as an important candidate for cell-based therapy in CVD. We hypothesized that MSCs derived from induced pluripotent stem cell (iPSC-MSCs) or bone marrow (BM-MSCs) might alleviate cigarette smoke (CS)-induced cardiac injury. This study aimed to investigate the effects of BM-MSCs or iPSC-MSCs on CS-induced changes in serum and cardiac lipid profiles, oxidative stress and inflammation as well as cardiac function in a rat model of passive smoking. Male Sprague-Dawley rats were randomly selected for exposure to either sham air (SA) as control or 4% CS for 1 h per day for 56 days. On day 29 and 43, human adult BM-MSCs, iPSC-MSCs or PBS were administered intravenously to CS-exposed rats. Results from echocardiography, serum and cardiac lipid profiles, cardiac antioxidant capacity, cardiac pro- and anti-inflammatory cytokines and cardiac morphological changes were evaluated at the end of treatment. iPSC-MSC-treated group showed a greater effect in the improvement of CS-induced cardiac dysfunction over BM-MSCs-treated group as shown by increased percentage left ventricular ejection fraction and percentage fractional shortening, in line with the greater reversal of cardiac lipid abnormality. In addition, iPSC-MSCs administration attenuated CS-induced elevation of cardiac pro-inflammatory cytokines as well as restoration of anti-inflammatory cytokines and anti-oxidative markers, leading to ameliorate cardiac morphological abnormalities. These data suggest that iPSC-MSCs on one hand may restore CS-induced cardiac lipid abnormality and on the other hand may attenuate cardiac oxidative stress and inflammation via inhibition of CS-induced NF-κB activation, leading to improvement of cardiac remodeling and dysfunction. Thus, iPSC-MSCs may be a promising candidate in cell-based therapy to prevent cardiac complications in smokers.

## Introduction

Cigarette smoking, one of the major risk factors for the development of cardiovascular disease (CVD), accounts for morbidity and mortality of cardiac events, including coronary heart disease, atherosclerosis, myocardial infarction and stroke (Burns, 2003). The association between CVD and the effect of passive (secondhand) smoking needs to be taken into consideration, as passive smoking increases the risk of coronary heart disease, myocardial infarction, stroke and cardiac death (Barnoya and Glantz, 2005; Minicucci et al., 2009). Despite evidence linking cigarette smoke (CS) exposure with CVD, the precise mechanism of CS-induced CVD remains largely unknown. The development of a rodent passive smoking model to study the early changes in CS-induced cardiac dysfunction would greatly help to delineate the pathophysiology of CVD. CS exerts its deleterious cardiovascular effects through several potential mechanisms involving oxidative stress, inflammation and modification of lipid profiles, leading to initiation and progression of atherothrombosis (Ambrose and Barua, 2004; Salahuddin et al., 2012). There is increasing evidence that smoking contributes to cardiac disorders is associated with lipid abnormality. CS exerts detrimental effect on the lipid metabolism, with elevation of serum cholesterol, triglycerides, and very low-density lipoprotein (VLDL)-cholesterol, along with reduction of serum high-density lipoprotein (HDL)-cholesterol and Apo lipoprotein A-I (Apo A-I) in smokers compared to non-smokers (Craig et al., 1989). Besides, elevation of plasma free fatty acids (FFA) levels was found in smokers compared to non-smokers (Hellerstein et al., 1994; Neese et al., 1994). Fatty acids are utilized by the heart to generate a constant supply of ATP needed for normal myocardial function (Stanley et al., 2005). Cardiac lipid profile also affects electrophysiological and mechanical function due to modulation of the physicochemical properties of cellular membranes (van der Vusse et al., 2000). However, the physiological and biochemical responses to CS have not been fully characterized in terms of cardiac lipid profile and function.

Nowadays, there is an increasing interest in the therapeutic potential of mesenchymal stem cells (MSCs) in CVD. MSCs rescued damaged hearts by reducing the scar size in clinical trials, ameliorated cardiac injury and improved ventricular function in animal models of acute and chronic myocardial infarction, as well as non-ischemia dilated cardiomyopathy (Gnecchi et al., 2012). In this study, a novel cell type of MSCs which derived from induced pluripotent stem cells (known as iPSC-MSCs) and bone marrow-derived MSCs (BM-MSCs) was intravenously administered to the CS-exposed rat model for comparison of their efficacies. BM-MSCs have limited capacity for proliferation and differentiation while iPSC-MSCs have unlimited resource, no need for immunosuppression, more consistency and high proliferative ability (expanded up to 40 passages) without loss of self-renewal capacity and constitutively express surface antigens of multipotent MSCs (Lian et al., 2010). Due to a lack of information available regarding the therapeutic potential of systemic delivery of iPSC-MSCs on CS-induced cardiac dysfunction, this study was aimed to explore the regulation of cardiac lipid metabolism after CS exposure and to compare the therapeutic potential of iPSC-MSCs over BM-MSCs on various outcomes including cardiac function, cardiac lipid profile, oxidative stress, inflammation and remodeling in a rat model of passive smoking.

## Materials and Methods

### Experimental Animals

Male Sprague-Dawley rats were purchased from and kept in the Laboratory Animal Unit of the University of Hong Kong, which is fully accredited by the Association for Assessment and Accreditation for Laboratory Animal Care (AAALAC International). All the animal experiments were performed in strict accordance with the recommendations in the Guide for the Care and Use of Laboratory Animals of the National Institutes of Health. All experimental procedures were approved by the Committee on the Use of Live Animal in Teaching and Research (CULATR, No.:2377-11) of the University of Hong Kong. The animals were kept at 22 ± 1°C, humidity (65∼75%) and day/night cycle (12/12-h light/dark).

### Preparation of iPSC-MSCs and BM-MSCs

Human iPSC-MSCs were prepared based on a previously described protocol (Lian et al., 2010). Characterized adult human BM-MSCs were purchased commercially (catalog No.: PT-2501, Cambrex Bioscience, Rockland, ME, United States). MSCs were cultured with DMEM plus 10% fetal calf serum (GIBCO, Carlsbad, CA, United States), basic fibroblast growth factor (bFGF, 5 ng/mL), and epidermal growth factor (EGF, 10 ng/mL). Details can be found in Supplementary Materials.

### CS-Exposed Rat Model

The passive smoking exposure used in this study was adapted from the original design as previously reported (Chan et al., 2009; Li et al., 2014). Briefly, rats (at 6 weeks of age, 160–200 g) were exposed to 4% CS, which produced comparable cotinine concentration to intermediate smokers (i.e., consumption of 11–20 cigarettes per day) for 1 h daily using commercially available cigarette [11 mg tar, 0.8 mg nicotine; (Camel; filter, R.J. Reynolds, Winston-Salem, NC, United States)] for 56 days in the ventilated chambers. All filters from cigarettes were removed before lighting up. The control rats underwent same procedure of exposure with fresh air.

### Groups and Treatment

Rats were divided into four groups, including sham air group (SA), CS group (CS), BM-MSCs treatment plus CS (BM-MSCs/CS) group and iPSC-MSCs treatment plus CS (iPSC-MSCs/CS) group, respectively. During the exposure period, two doses of BM-MSCs or iPSC-MSCs (3 × 10^6^ cells in 0.5 ml) were injected intravenously (*i.v.*) via tail vein to the BM-MSCs/CS or iPSC-MSCs/CS group on day 29 and day 43, while other groups (SA and CS groups) were injected with phosphate-buffered saline (PBS) of the same volume. All rats were underwent echocardiography and sacrificed 24 h after the last CS exposure (Supplementary Figure S1).

### Echocardiographic Analysis

Echocardiography was conducted on all rats in the supine position under anesthesia with ketamine (70 mg/kg body weight)/xylazine (6 mg/kg body weight) (*i.p.*) by using a high resolution Micro-Ultrasound System (Vevo^TM^ 770, Visual Sonics Inc., Toronto, ON, Canada) equipped with a 25-MHz linear transducer before termination of the experiment. Bi-dimensional echocardiographic variables were measured from M-mode images, including left ventricular end diastolic dimension (LVEDD), left ventricular end systolic dimension (LVESD), left ventricular end diastolic posterior wall dimension (LVPWd) and left ventricular end systolic posterior wall dimension (LVPWs). Left ventricular systolic function was accessed by calculating percentage left ventricular ejection fraction (%LVEF) and percentage fractional shortening (%FS). After the echocardiographic measurement, rats were euthanized with pentobarbital at a lethal dose (100 mg/kg body weight). Blood and heart tissues were collected for further study.

### Preparation of Total Protein and Nuclear/Cytoplasmic Protein Fraction

Frozen heart tissue (without the atria) was ground up with a mortar and pestle under liquid nitrogen. For total protein extraction, heart tissue was homogenized in T-PER^®^ tissue protein extraction reagent containing protease/phosphatase inhibitors cocktail (Thermo Scientific, Rockford, IL, United States) and 100 mM Phenylmethanesulfonyl fluoride (PMSF) following the manufacturer’s instruction. The extraction of nuclear/cytoplasmic protein was performed using NE-PER^®^ nuclear and cytoplasmic extraction reagents (Thermo Scientific, Rockford, IL, United States) according to manufacturer’s instruction. Protein concentrations were measured by Bradford protein assay (Bio-Rad Laboratories, Philadelphia, PA, United States) using bovine serum albumin (BSA) as standards.

### Lipid Extraction and Measurement

Lipid extraction from the frozen heart tissue was adapted and carried out according to the established protocol (Zou, 2011). Briefly, heart tissues were weighted (about 20 mg) and put into a 15 ml tube, 4 ml lipid extraction buffer (2 volume chloroform and 1 volume methanol) was added into the tube. After vigorously vortex, the tissue was incubated for 16 h at 4°C. Two milliliters 0.6% sodium chloride was added and the mixture was centrifuged at 2,000 × *g* for 20 min. The organic layer (lower layer) of the liquid was collected and was evaporated the organic solvent by using Savant^TM^ SC110A Speed Vac Plus concentrator with Universal Vacuum System Plus (Thermo Scientific). The pellet was dissolved with lipid dissolution buffer (5 volume isopropanol, 2 volume water and 2 volumes of Triton X-100) for the measurement of lipid parameters. Levels of cholesterol and triglyceride in the serum and heart lipid extract were determined with kits from Stanbio Laboratory (Cat #1010 and 2100; Boerne, TX, United States) according to manufacturer’s instructions. Serum and cardiac FFA levels were determined with half-micro test kit from Roche (Cat #11-383-175-001, Roche Applied Science, Mannheim, Germany). Levels of lipid parameters in heart homogenate were corrected by the wet weight of heart tissue used in the lipid extraction.

### Determination of Oxidative Stress and Pro-/Anti-inflammatory Markers

Total anti-oxidant capacity (T-AOC) and enzyme activity of total superoxide dismutase (SOD) were examined by kits (Nanjing Jiancheng Bioengineering Institute, China) and activity of catalase (CAT) was determined by Amplex^®^ Red catalase assay kit (Molecular Probes Inc., Invitrogen, Eugene, OR, United States). Commercial Enzyme-linked immunosorbent assay (ELISA) kits were used to measure the levels of cytokine-induced neutrophil chemoattractant-1 (CINC-1, resembles to human IL-8; R&D Inc., Minneapolis, MN, United States), TNF-α (eBioscience, San Diego, CA, United States), IL-10 (BD Biosciences, San Diego, CA, United States), and adiponectin (Invitrogen, Eugene, OR, United States) in rat heart homogenates according to the manufacturer’s instructions. The absorbance was measured by a microplate-reader and the results were corrected by heart protein concentration.

### Western Blot Analysis

Equal amounts of heart protein extractions (40 μg) were separated in 10% SDS–PAGE gel and transferred onto a nitrocellulose membrane (0.45 μm, Amersham Hybond ECL, GE Healthcare, Germany). After blocking, membranes were incubated with diluted specified primary antibodies, including NF-κB p65, IκBα (1:1000, catalog No.: #4764 and #4814, Cell Signaling), ATP-binding cassette protein-A1 (ABCA1) (1:1000, catalog No.: NB400-105, Novus Biologicals, Littleton, CO, United States), fatty acid translocase (FAT)/CD36 (CD36), fatty acid synthase (FAS), 3-hydroxy-3-methylglutaryl coenzyme A reductase (HMGCR) or low density lipoprotein receptor (LDL-R) (1:500, catalog No.: NB110-59724, NB400-114, NBP1-50713 and NB110-57162, Novus Biologicals), lipoprotein lipase (LPL) (1:200, catalog No.: sc-373759, Santa Cruz Biotechnology, Inc., Dallas, TX, United States) at 4°C overnight. Before developing with an enhanced chemiluminescence (ECL) reagent (Amersham, Piscataway, United Kingdom), membranes were incubated with the appropriate horseradish peroxidase (HRP)-conjugated secondary antibodies (Dako, Denmark). Bands were visualized on medical X-ray film (Fumingwei, Shenzhen, China). After stripping, membranes were re-probed for glyceraldehyde 3-phosphate dehydrogenase (GAPDH) or Lamin A/C as loading control. Densitometric analysis of the bands was performed with Gene Tools (Syngene), and results were expressed as fold change to relative control.

### Histology

Hematoxylin and eosin (H&E) for morphological changes and Sirius red staining for fibrotic detection were performed on formalin-fixed paraffin-embedded heart sections (5 μm). Transverse sections were captured digitally, and acquired by using a Zeiss Axioskop 2 plus microscope (Zeiss, Göttingen, Germany). Cardiomyocyte cross-sectional area was measured using the Axio Vision Rel. 4.6 software (Zeiss). We traced the outline of at least 100 cardiomyocytes per heart, and the data were averaged. Quantification of the Sirius red staining was determined as positive area in any five fields per heart section and analysis was performed with the help of Image J (NIH) with additional threshold color plug-ins to process the *tiff* file images. Images were analyzed by two independent and blinded investigators.

### Statistical Analysis

All results were expressed as mean ± SEM for indicated number of animals (n). The comparison among multiple groups was performed by one-way analysis of variance (ANOVA) followed by the *post hoc* Bonferroni’s test. Differences were considered to be significant when the two-sided *p*-value was less than 0.05. All statistical analyses were performed using computer software (Prism 5.0, GraphPad Software, San Diego, CA, United States).

## Results

### iPSC-MSCs Attenuated CS-Induced Cardiac Dysfunction

The data from echocardiographic study are presented in **Table 1**. The CS-exposed rats (CS group) developed cardiac contractile dysfunction that was characterized by significantly decreased %LVEF and %FS when compared with SA-exposed rats (SA group) (*p* < 0.01, respectively), which was significantly restored to a normal status in the presence of iPSC-MSCs (iPSC-MSCs/CS group; *p* < 0.05) but not BM-MSCs. There was also a significant increase of LVESD and a significant decrease of LVPWs in the CS group compared to the SA group (*p* < 0.05, respectively), suggesting a possibility of morphological changes, which further led to functional changes observed in CS-induced reduction of %LVEF and %FS.

**Table 1:** Echocardiographic study.

			BM-	iPSC-
	SA	CS	MSCs/CS	MSCs/CS
LVEDD (mm)	7.95 ± 0.32	8.40 ± 0.23	8.04 ± 0.25	8.45 ± 0.24
LVESD (mm)	4.57 ± 0.43	5.61 ± 0.16^∗^	5.53 ± 0.24	5.16 ± 0.17
LVPWd (mm)	1.90 ± 0.11	1.79 ± 0.10	1.85 ± 0.06	1.89 ± 0.06
LVPWs (mm)	2.96 ± 0.15	2.47 ± 0.07^∗^	2.63 ± 0.16	2.74 ± 0.08
LVEF (%)	78.33 ± 3.27	67.14 ± 1.75^∗∗^	68.43 ± 1.97	74.57 ± 0.97^#^
FS (%)	43.17 ± 3.61	33.29 ± 1.15^∗∗^	34.43 ± 1.49	39.00 ± 0.81^#^

*LVEDD, left ventricular end diastolic dimension; LVESD, left ventricular end systolic dimension; LVPWd, left ventricular end diastolic posterior wall dimension; LVPWs, left ventricular end systolic posterior wall dimension; LVEF, left ventricular ejection fraction; FS, fractional shortening. Results are expressed as mean ± SEM; SA, *n* = 6; CS, *n* = 7; BM-MSCs/CS, *n* = 7; iPSC-MSCs/CS, *n* = 7. Data were analyzed by one-way ANOVA. ^∗^*P* < 0.05, ^∗∗^*P* < 0.01 or comparison between the CS group and SA group. ^#^*P* < 0.05 for comparison between iPSC-MSCs/CS or BM-MSCs/CS group and CS group.*

### iPSC-MSCs Restored Cardiac But Not Serum Lipid Abnormality

The serum and cardiac lipid profiles are presented in **Table 2**. There were no significant differences between groups in serum cholesterol and triglyceride. Serum FFA level showed a trend of elevation in the CS group compared to the SA group but not reaching significant level, which was not reversed in the presence of either BM-MSCs or iPSC-MSCs.

**Table 2:** Effects of MSCs on serum and cardiac lipid profiles.

	SA	CS	BM-MSCs/CS	iPSC-MSCs/CS
**Serum lipid profile**				
Cholesterol (mg/dL)	60.98 ± 3.78	66.86 ± 2.66	62.20 ± 1.84	59.50 ± 1.64
Triglycerides (mg/dL)	138.7 ± 12.4	155.0 ± 15.8	146.2 ± 28.5	146.8 ± 11.3
FFA (μM)	74.03 ± 6.02	111.0 ± 22.5	120.7 ± 17.9	117.3 ± 18.73
**Cardiac lipid profile**				
Cholesterol (mg/g tissue)	2.338 ± 0.10	2.726 ± 0.10^∗^	2.406 ± 0.11	2.317 ± 0.10^#^
Triglycerides (mg/g tissue)	4.842 ± 0.13	6.517 ± 0.54^∗∗^	4.986 ± 0.27^#^	5.119 ± 0.22^#^
FFA (μ mole/g tissue)	1.727 ± 0.14	1.160 ± 0.08^∗∗^	1.241 ± 0.09	1.550 ± 0.12^#^

*Data are expressed as mean ± SEM; *n* = 6–8 in each group. Data were analyzed by one-way ANOVA. ^∗^*P* < 0.05, ^∗∗^*P* < 0.01 or comparison between the CS and SA groups. ^#^*P* < 0.05 for comparison between the iPSC-MSCs/CS or BM-MSCs/CS groups and CS group. FFA, free fatty acid.*

Cigarette smoke exposure significantly elevated the cardiac levels of cholesterol and triglyceride (*p*< 0.05 and *p*< 0.01, respectively), which was attenuated after BM-MSCs or iPSC-MSCs treatment for cardiac triglyceride (*p* < 0.05, respectively) while only iPSC-MSCs treatment (iPSC-MSCs/CS group) significantly reversed cardiac cholesterol (*p*< 0.05). On the contrary, CS caused a significant reduction in cardiac FFA level (*p*< 0.01), which was augmented only in iPSC-MSCs/CS group (*p*< 0.05).

### iPSC-MSCs Regulated Cardiac Cholesterol Metabolism-Related Proteins

**FIGURE 1:**
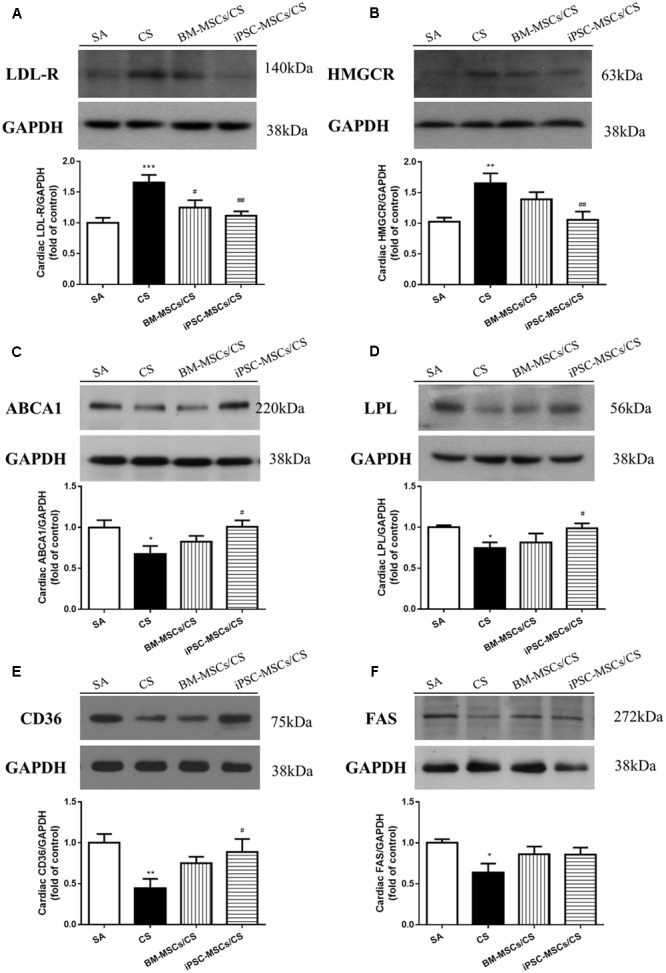
Effect of MSCs on cardiac expressions of proteins regulating cholesterol, triglycerides and FFA metabolism. **(A)** CS exposure caused significant elevation of cardiac expression of LDL-R, which was attenuated in both the BM-MSCs/CS and iPSC-MSCs/CS groups. **(B)** CS led to increased cardiac expression of HMGCR, which was attenuated in the iPSC-MSCs/CS group. **(C)** CS reduced cardiac expression of ABCA1, which was restored in the iPSC-MSCs/CS group. **(D)** CS exposure significantly reduced protein expression of LPL, which was restored in the iPSC-MSCs/CS group. **(E)** CS exposure led to a significant reduction in protein expression of cardiac CD36, which was significantly restored in the iPSC-MSCs/CS group. **(F)** Cardiac FAS protein expression was inhibited after CS exposure, which showed a trend of restoration but not reaching significant level in both the BM-MSCs/CS and iPSC-MSCs/CS groups. Cardiac levels of protein expression were normalized to GAPDH levels and expressed as fold change vs. relative control. Data are expressed as mean ± SEM; *n* = 7–8. Data were analyzed by one-way ANOVA. ^∗^*p* < 0.05, ^∗∗^*p* < 0.01, ^∗∗∗^*p* < 0.001 for comparison between the CS and SA groups. ^#^*p* < 0.05, ^##^*p* < 0.01 for comparison between the BM-MSCs/CS or iPSC-MSCs/CS groups and CS group, respectively. LDL-R, low density lipoprotein receptor; GAPDH, glyceraldehyde 3-phosphate dehydrogenase; HMGCR, 3-hydroxy-3-methylglutaryl coenzyme A reductase; ABCA1, ATP-binding cassette protein-A1; LPL, Lipoprotein lipase; CD36, fatty acid translocase (FAT)/CD36; FAS, fatty acid synthase.

Cigarette smoke exposure caused elevated protein expressions of LDL-R and HMGCR (*p*< 0.001 and *p*< 0.01, respectively), mediating cholesterol influx and synthesis, and decreased protein expression of ABCA1 (*p*< 0.05), controlling the efflux of cholesterol. The reversal of CS-induced up-regulation of LDL-R protein expression was observed in both the BM-MSCs/CS and the iPSC-MSCs/CS groups (*p*< 0.05 and *p*< 0.01, respectively). However, the restoration of CS-induced up-regulation of HMGCR protein expression was found to be significant only in iPSC-MSCs/CS group (*p*< 0.01). In addition, the CS-induced down-regulation of ABCA1 protein expression was significantly restored only in the iPSC-MSCs/CS group (*p*< 0.05) (**Figures 1A–C**).

### iPSC-MSCs Regulated Cardiac Triglycerides Metabolism-Related Proteins

Protein expression of cardiac lipid-metabolizing enzyme LPL was significantly decreased in CS-exposed group compared to the SA-exposed group (*p* < 0.05), which was restored only in the iPSC-MSCs/CS group (*p* < 0.05) (**Figure 1D**).

### iPSC-MSCs Regulated Cardiac Fatty Acid Metabolism-Related Proteins

Cigarette smoke exposure led to a significant reduction in protein expression of cardiac CD36 and FAS (*p*< 0.01 and *p*< 0.05, respectively). The restoration of CS-induced reduction in cardiac CD36 protein expression reached significant level only in the iPSC-MSCs/CS group (*p*< 0.05) (**Figure 1E**). However, the CS-induced suppression of cardiac FAS protein expression was found to show a trend of reversal but not reaching significance in both iPSC-MSCs/CS and BM-MSCs/CS groups (**Figure 1F**).

### iPSC-MSCs Attenuated Cardiac CS-Induced Oxidative Stress

**FIGURE 2:**
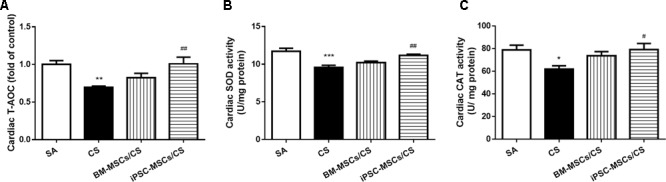
Effect of MSCs on cardiac levels of oxidative stress markers. Cardiac T-AOC **(A)**, SOD activity **(B)** and CAT activity **(C)** were reduced after CS exposure, which were significantly restored in the iPSC-MSCs/CS group. Data are expressed as mean ± SEM; *n* = 7–8. Data were analyzed by one-way ANOVA. ^∗^*p* < 0.05, ^∗∗^*p* < 0.01, ^∗∗∗^*p* < 0.001 for comparison between the CS and SA groups. ^#^*p* < 0.05, ^##^*p* < 0.01 for comparison between the BM-MSCs/CS or iPSC-MSCs/CS group and the CS group, respectively. T-AOC, total anti-oxidant capacity; SOD, superoxide dismutase; CAT, catalase.

As an overall anti-oxidative marker, cardiac T-AOC was significantly decreased in CS group (*p* < 0.01), which was restored only in the iPSC-MSCs/CS group (*p* < 0.01) (**Figure 2A**). The enzyme activities of cardiac SOD and CAT were significantly inhibited in the CS group compared to the SA group (*p* < 0.001 and *p* < 0.05, respectively), which was significantly restored only in the iPSC-MSCs/CS group (*p* < 0.01 and *p* < 0.05, respectively) (**Figures 2B,C**).

### iPSC-MSCs Regulated Cardiac Pro-/Anti-inflammatory Mediators

**FIGURE 3:**
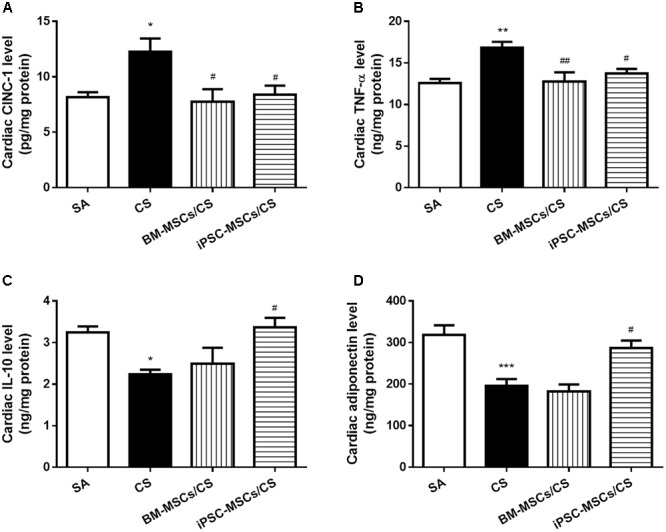
Effect of MSCs on cardiac levels of inflammatory markers. Cardiac levels of pro-inflammatory cytokines CINC-1 **(A)** and TNF-α **(B)** were elevated after CS exposure, which were reserved in both the BM-MSCs/CS and iPSC-MSCs/CS groups. Cardiac levels of anti-inflammatory markers IL-10 **(C)**, adiponectin **(D)** were reduced in the CS group, which was reversed in the iPSC-MSCs/CS group. Data are expressed as mean ± SEM; *n* = 7–8. Data were analyzed by one-way ANOVA. ^∗^*p* < 0.05, ^∗∗^*p* < 0.01, ^∗∗∗^*p* < 0.001 for comparison between the CS and SA groups. ^#^*p* < 0.05, ^##^*p* < 0.01 for comparison between the BM-MSCs/CS or iPSC-MSCs/CS group and the CS group, respectively. CINC-1, cytokine-induced neutrophil chemoattractant-1; TNF-α, tumor necrosis factor-α; IL-10, interleukin-10.

Cardiac levels of pro-inflammatory markers, including CINC-1 and TNF-α, were significantly elevated in the CS group compared to the SA group (*p* < 0.05 and *p* < 0.01, respectively). Both BM-MSCs/CS and iPSC-MSCs/CS groups attenuated CS-induced elevations of all these pro-inflammatory mediators (*p* < 0.01 for comparison between CS group and BM-MSCs/CS group in the level of TNF-α, and *p* < 0.05 for others) (**Figures 3A,B**). On the other hand, anti-inflammatory markers, both interleukin-10 (IL-10) and adiponectin, were significantly reduced in the CS group compared to the SA group (*p* < 0.05 and *p* < 0.001, respectively), which were significantly reversed only in iPSC-MSCs/CS group (*p* < 0.05, respectively) (**Figures 3C,D**).

### iPSC-MSCs Inhibited Cardiac CS-Induced NF-κB Activation

**FIGURE 4:**
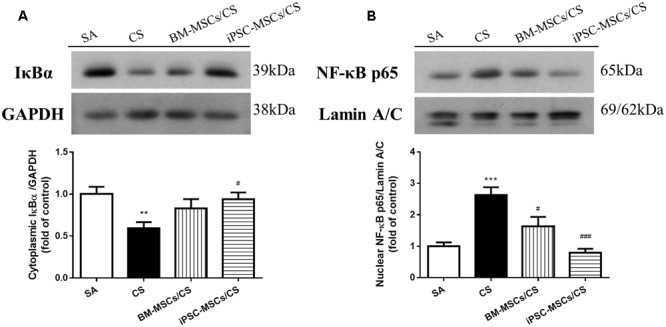
Effect of MSCs on protein expressions of NF-κB signaling pathway in the heart. **(A)** Protein expression level of cardiac cytoplasmic IκBα was normalized to GAPDH levels. CS exposure reduced cardiac cytoplasmic IκBα expression level, which was reversed in iPSC-MSCs/CS group. **(B)** Protein expression level of cardiac nuclear NF-κB p65 was normalized to Lamin A/C levels. CS exposure increased NF-κB p65 protein expression in the nuclear fraction of the heart, which was attenuated in both the BM-MSCs/CS and iPSC-MSCs/CS groups. Results are expressed as mean ± SEM; *n* = 7–8. Data were analyzed by one-way ANOVA. ^∗∗^*p* < 0.01, ^∗∗∗^*p* < 0.001 for comparison between the CS and SA groups. ^#^*p* < 0.05, ^###^*p* < 0.001 for comparison between the BM-MSCs/CS or iPSC-MSCs/CS groups and CS group, respectively. NF-κB, nuclear factor-κB.

Due to the major contributing role in oxidative and inflammatory responses, we further investigated the effects of iPSC-MSCs and BM-MSCs on the NF-κB signaling pathway, including protein expression of cardiac cytoplasmic IκBα and nuclear NF-κB p65. CS exposure caused a significant reduction in the levels of cardiac cytoplasmic IκBα expression (*p* < 0.01), indicating proteasomal degradation as a key step for NF-κB activation, and a significant elevation of cardiac nuclear NF-κB p65 (*p* < 0.001), suggesting nuclear translocation in the CS group compared to the SA group (**Figures 4A,B**). iPSC-MSCs treatment significantly reversed both CS-induced reduction of IκBα (*p* < 0.05) and CS-induced upregulation of NF-κB p65 (*p* < 0.001), but not BM-MSCs treatment (**Figures 4A,B**).

### iPSC-MSCs Attenuated CS-Induced Cardiac Hypertrophy and Interstitial Fibrosis

**FIGURE 5:**
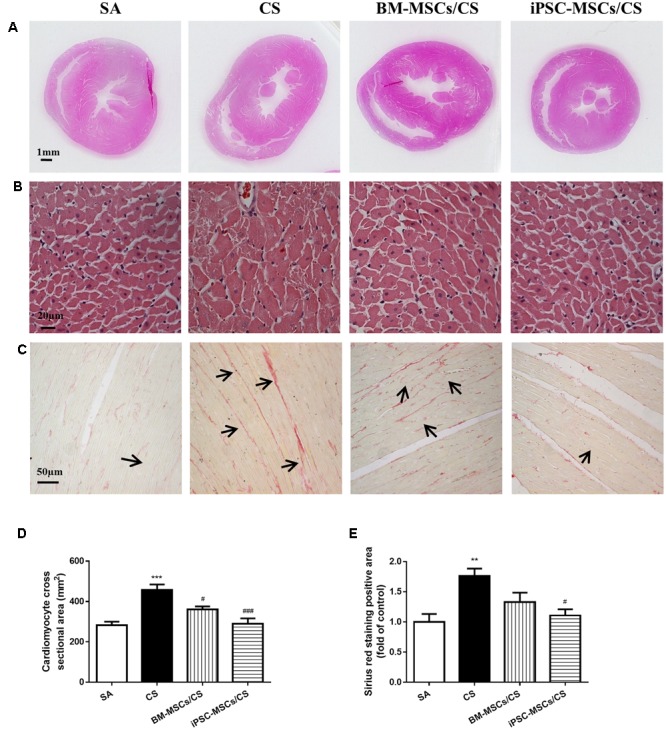
Effect of MSCs on cardiac morphological alterations. **(A)** Representative photographs show obvious gross differences in transverse ventricular sections among rats who received different treatments. **(B)** Representative photomicrographs of cardiomyocytes (magnification, ×400; scale bar = 20 μm). **(C)** Representative photomicrographs of myocardial interstitial fibrosis in heart sections stained with Sirius red (magnification, ×200, scale bar = 50 μm). **(D)** Quantitative analysis of cardiomyocyte cross-sectional area from at least 100 cardiomyocytes per heart. **(E)** Quantification of the Sirius red staining was determined as positive area in five random fields per heart section and analysis was performed using Image J software. Results are expressed as mean ± SEM; *n* = 4–6. Data were analyzed by one-way ANOVA. ^∗∗^*p* < 0.01, ^∗∗∗^*p* < 0.001 for comparison between the CS and SA groups. ^#^*p* < 0.05 and ^###^*p* < 0.05 for comparison between the BM-MSCs/CS or iPSC-MSCs/CS groups and the CS group, respectively.

Extensive cardiac remodeling was observed after 56-day CS exposure, characterized by elongation of the heart shape in the gross histological analysis (**Figure 5A**). Microscopic observation on heart sections further revealed that the cross sectional area of cardiomyocytes in the left ventricle was significantly increased (i.e., hypertrophy) in the CS group compared to the SA group (*p* < 0.001). Treatment with either BM-MSCs or iPSC-MSCs significantly attenuated CS-induced cardiac hypertrophy in both the BM-MSCs/CS and iPSC-MSCs/CS groups (*p* < 0.05 and *p* < 0.001, respectively) (**Figures 5B,D**).

To confirm the presence of the interstitial fibrosis, Sirius red staining was performed on heart sections (**Figure 5C**). In line with the cardiac function, increased cardiac interstitial fibrosis was observed in the CS group compared to the SA group (*p* < 0.01), which was significantly attenuated only in the iPSC-MSCs/CS group (*p* < 0.05) (**Figure 5E**).

## Discussion

In this study, we found that the smoking exposure resembling passive smokers led to contractile dysfunction and cardiac hypertrophy, and the functional compromise was accompanied by interstitial fibrosis. iPSC-MSCs were found to be more effective than BM-MSCs in restoring CS-induced cardiac dysfunction via regulation of cholesterol, triglyceride and fatty acid metabolism, attenuation of cardiac inflammation and oxidative stress as well as amelioration of cardiac remodeling. The mechanism might partly involve inhibiting the NF-κB signaling pathway.

The findings of smoking effect on cardiac dysfunction characterized by lower LVEF and FS was in agreement with a previous report (Gu et al., 2008). The beneficial effect of MSCs in improving CS-induced cardiac dysfunction was similar to previous clinical report with the use of MSCs in patients with acute myocardial infarction or chronic ischemic cardiomyopathy (Karantalis and Hare, 2015).

Lipids play important roles in virtually all aspects of biological life. Cardiac lipid affects electrophysiological and mechanical function due to modulation of the physicochemical properties of cellular membranes (van der Vusse et al., 2000). In this study, the elevation of CS-induced cardiac cholesterol and triglyceride levels was in line with the previous studies (Latha et al., 1988; Gokulakrisnan et al., 2011), suggesting that cardiac damage might be due to the excessive accumulation of cardiac cholesterol and triglyceride.

Cholesterol plays an essential role in maintaining the structure of cell membranes; however, excessive cholesterol accumulation in cells induces cytotoxicity (Tabas, 2002). The cholesterol homeostasis within the cell is regulated by endogenous synthesis mainly by a rate-limiting enzyme HMGCR (Eisa-Beygi et al., 2014), receptor-mediated endocytosis by LDL-R on the cell surface and the efflux of cholesterol (Tabas, 2002). During the efflux process of excessive cholesterol, ABCA1 initiates HDL formation (Tang and Oram, 2009) as the primary gatekeeper in cellular cholesterol removal (Oram and Lawn, 2001; Oram and Vaughan, 2006; Tang and Oram, 2009). CS-induced elevation in cardiac cholesterol level was due to the increased cholesterol biosynthesis, increased uptake from blood stream and the decreased efflux of cholesterol through the elevation of HMGCR and LDL-R, and the reduction of ABCA1 protein expression, respectively. These direct actions of high cholesterol levels on cardiomyocytes, vascular smooth muscle cells and endothelium result in cardiac dysfunction by affecting contractility, excitability and conduction properties (Saini et al., 2004) and cardiac hypertrophy (Shmeeda et al., 1994), in line with the current findings. iPSC-MSCs over BM-MSCs treatment showed a greater capacity in overall reduction in cardiac cholesterol level through restoration of LDL-R, HMGCR and ABCA1 expressions, suggesting that the contribution of the inhibition of synthesis and influx, and the promotion of efflux of cholesterol may restore CS-induced cardiac remodeling and dysfunction.

Heart is the most energy demanding organ in the body, which requires constantly high energy supply to sustain the continuous contractile activity. Most of the cardiac energy metabolism relies on the oxidation of FA (Stanley et al., 2005). In general, FA are taken up by the cardiomyocytes from either plasma FA bound to albumin or from FA contained within chylomicron or VLDL triacylglycerol via the direct passive membrane diffusion or carrier-mediated proteins (Lopaschuk et al., 2010). Among these transporters of FA, FAT/CD36 contributes to 50–60% FA uptake, exerting its key role in the regulation of cardiac FA metabolism (Lopaschuk et al., 2010). FAS, an important multi-enzyme protein, mediates fatty acid synthesis to maintain cardiac function during stress (Razani et al., 2011). Another functional enzyme LPL which can be synthesized in cardiomyocytes, is to catalyze the breakdown of the triglycerides component of lipoproteins and the hydrolysis of circulating triglyceride-rich lipoproteins to provide FA to the heart (Augustus et al., 2003). In this study, CS exposure caused a reduction of cardiac FFA in line with the reduction of cardiac CD36 expression as the uptake and oxidation of FA was limited. The CS-induced reduction of cardiac FAS indicated the reduction of *de novo* synthesis, in agreement with a previous study (Razani et al., 2011). The elevation of cardiac triglyceride level and reduction of cardiac FFA may be explained by the marked reduction of LPL expression, indicating the impaired triglycerides clearance. Loss of LPL-derived lipids leads to decreased triglyceride lipolysis and increased glucose uptake in heart, resulting in cardiac dysfunction (Augustus et al., 2003, 2006; Khan et al., 2013). Although neither BM-MSCs nor iPSC-MSCs treatment could alter the serum FFA level, treatment with iPSC-MSCs restored cardiac FFA level, in support of the reversal of cardiac CD36 expression after treatment with iPSC-MSCs, which accounts for over 50% FA uptake in the heart.

Oxidative stress, the imbalance between oxidants and antioxidants, plays an important role in cardiac function (Hori and Nishida, 2009; Tsutsui et al., 2011). ROS activates a broad variety of transcription factors such as NF-κB, and eventually influences cardiac contractile function by oxidizing proteins involved in excitation-contraction coupling (Tsutsui et al., 2011). In this study, CS-induced oxidative stress was characterized by impaired total antioxidant capacity in the heart, which was restored in the presence of iPSC-MSCs, in agreement with acute lung injury and stroke models, indicating anti-oxidative capacity of MSCs (Calio et al., 2014; Shalaby et al., 2014). CS-induced oxidative stress appears to be at the initial stage before triggering inflammatory responses due to excessive ROS production (Das et al., 2012), which would have a great influence on cardiac function (Frangogiannis, 2012). In line with previous findings (Guo et al., 2007; Du et al., 2008), CS caused up-regulation of pro-inflammatory cytokines including CINC-1 (a homolog to human IL-8) and TNF-α, which was attenuated in the treatment of either BM-MSCs or iPSC-MSCs. On the other hand, CS down-regulated anti-inflammatory markers including IL-10 and adiponectin, which was restored only after iPSC-MSCs treatment, suggesting the superior capacity of iPSC-MSCs over BM-MSCs in alleviating inflammation. The anti-inflammatory cytokine IL-10, one of the major modulator of pro-inflammatory cytokines/chemokines, prevented TNF-α-induced activation of NF-κB in cardiomyocytes (Dhingra et al., 2009). Adiponectin, an adipokine secreted mainly in adipose tissues and cardiomyocytes, was found to be at low circulating level in CVD (Shibata et al., 2009). Suppression of TNF-α and induction of IL-10 was found to be caused by adiponectin (Qi et al., 2014; Watanabe et al., 2014). In a previous report, cardiac generation of adiponectin had the protective effect against ischemia/reperfusion injury and against cardiomyocyte injury *in vivo* (Wang et al., 2010). In addition, adiponectin was shown to have an anti-hypertrophic effect in cardiomyocytes (Shibata et al., 2004).

Cigarette smoke-induced reduction of cardiac cytoplasmic IκBα levels and upregulation of cardiac nuclear NF-κB p65 levels were in line with previous reports (Gokulakrisnan et al., 2011; Das et al., 2012), confirming the involvement of NF-κB activation. Activation of nuclear factor-κB (NF-κB), a redox-sensitive transcription factor, leads to increased production of inflammatory cytokines, the key mediators in the initiation and progression of cardiac damage (Gordon et al., 2011). In agreement with previous publications (Du et al., 2008; Wang et al., 2013), we found the restoration of IκBα expression in the cytoplasm and the reduction in NF-κB p65 expression in the nuclei after iPSC-MSCs treatment. These results indicated that iPSC-MSCs treatment could effectively inhibit the activation of NF-κB and thus attenuate CS-induced oxidative stress and imbalance of pro-/anti-inflammatory mediators in the heart.

During cardiac remodeling process, hypertrophy is normally a compensatory response at the initiation of cardiac damage, leading to morphological adaptation to preserve the cardiac function. However, we observed cardiac dysfunction after 8-week CS exposure, which may be due to prolonged cardiac remodeling, in agreement with a previous study (Gu et al., 2008). In this study, attenuation of CS-induced cardiomyocyte hypertrophy and interstitial fibrosis in the presence of iPSC-MSCs might restore cardiac function, similar to a rat model of post-ischemic heart failure (Mias et al., 2009).

Despite unraveling some novel findings on the therapeutic effect of iPSC-MSCs, several limitations pose the need for further investigations in the future. Firstly, the current exposure regimen of passive smoking for 8 weeks in animals may not reflect the human scenario in active smokers. Secondly, we did not fully explore the mechanistic insight how MSCs exert their actions on lipid metabolism and the anti-oxidative, anti-inflammatory, anti-hypertrophic and anti-fibrotic effects, except the involvement of NF-κB signaling pathway. In addition, due to the detection of small number of MSCs present in the cardiac tissue (Supplementary Figure S2) after treatment, paracrine effect might be the possible mechanism of MSCs in the amelioration of CS-induced cardiac injury. In our previous study, we found that the secretions from iPSC-MSCs contained higher levels of macrophage migration inhibitory factor (MIF), growth differentiation factor-15 (GDF-15) and stem cell factor (SCF) than those from BM-MSCs, which might be responsible for some of the anti-oxidative, anti-inflammatory and anti-apoptotic effects of iPSC-MSCs (Zhang et al., 2015; Li et al., 2017). Nevertheless, iPSC-MSCs treatment capable of reducing CS-mediated cardiac dysfunction may be relevant to human health. This study highlighted changes in lipid profile that could potentially affect cardiac function. However, further studies are warranted to provide more comprehensive information by exploring specific pathways relevant to these changes in the rat model of passive smoking.

## Conclusion

**FIGURE 6:**
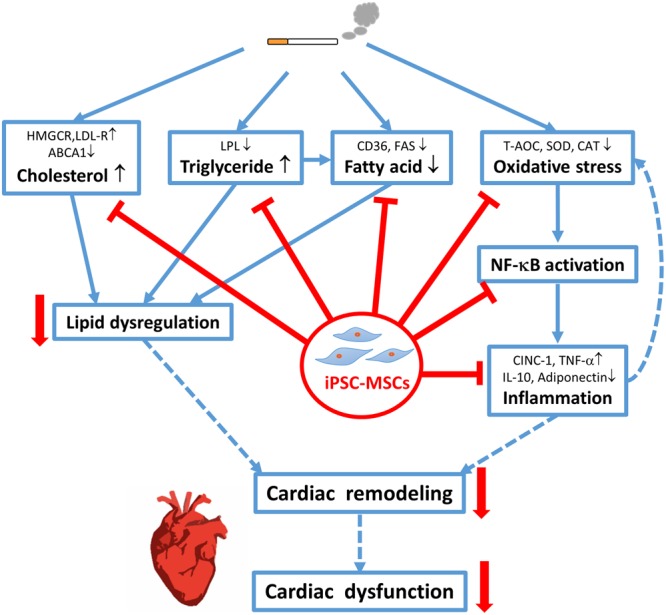
Schematic diagram. Cigarette smoke exposure causes cardiac lipid dysregulation and oxidative stress-induced cardiac inflammation that leads to morphological alterations (i.e., cardiac remodeling), resulting in cardiac dysfunction. Treatment with iPSC-MSCs counteracts the CS-induced cardiac remodeling and dysfunction through the restoration of cardiac lipid anomalies and attenuation of cardiac oxidative stress and inflammation via NF-κB signaling pathway. iPSC-MSCs, induced pluripotent stem cells-derived mesenchymal stem cells. NF-κB, Nuclear factor-kappa B. Solid lines indicate the presented data in this study while dashed lines indicate the causal relationship that needs further investigation.

Taken together, this study demonstrated a higher capacity of iPSC-MSCs over BM-MSCs in improving the CS-induced cardiac remodeling and dysfunction through the possible mechanisms of the restoration of cardiac lipid abnormality as well as the attenuation of cardiac oxidative stress, inflammation via inhibition of NF-κB activation (**Figure 6**). These findings indicate that iPSC-MSCs may be a promising candidate in cell-based therapy to prevent cardiac complications in smokers.

## Ethics Statement

This article does not contain any studies with human participants performed by any of the authors. All procedures performed in studies involving animals were in accordance with the ethical standards of the Committee on the Use of Live Animal in Teaching and Research (CULATR) in the University of Hong Kong.

## Author Contributions

YL conceived, performed and interpreted the experiments and wrote the draft of the manuscript. XL conducted the animal study and helped with sample preparation. YZ conceived and provided MSCs. SCY helped with the animal study and analyzed the data. ZZ performed the echocardiographic study on the animals. MSI helped with the experimental design and editing the manuscript. HFT provided experimental advice, manuscript editing. QL helped with the experimental design and editing manuscript. JCM conceived, interpreted the experiments and assisted with editing the manuscript. All authors reviewed and approved the manuscript.

## Conflict of Interest Statement

The authors declare that the research was conducted in the absence of any commercial or financial relationships that could be construed as a potential conflict of interest.
