# Supplementary material for: Induced Pluripotent Stem Cells-Derived Mesenchymal Stem Cells Attenuate Cigarette Smoke-Induced Cardiac Remodeling and Dysfunction
Source: Front Pharmacol. 2017 Jul 28;8:501. doi: 10.3389/fphar.2017.00501 (PMC5532447; doi:10.3389/fphar.2017.00501)
Supplement: Supplementary file 1 [file Data_Sheet_1.pdf]

Supplementary Material

Induced Pluripotent Stem Cells-Derived Mesenchymal Stem Cells Attenuate Cigarette Smoke-Induced Cardiac Remodeling and Dysfunction

iPSC-MSCs Attenuate Smoking-Induced Cardiac Dysfunction

Yingmin Liang, Xiang Li, Yuelin Zhang, Sze Chun Yeung, Zhe Zhen, Mary S. M. Ip, Hung Fat Tse, Qizhou Lian, Judith C. W. Mak

SUPPLEMENTAL MATERIALS AND METHODS

Preparation of iPSC-MSCs and BM-MSCs

Human iPSC-MSCs were prepared by Professor Tse's group from Cardiology Division, Department of Medicine, Li Ka Shing Faculty of Medicine, The University of Hong Kong. Based on a previously described protocol, iMR90 fibroblast cells (Cat# CCL-186, American Type Culture Collection, Manassas, VA, USA) were transduced with lentivirus-mediated Oct4, Sox2, Nanog and Lin28 factors by plasmid (Addene, Cat #16577-80, Cambridge, MA, USA) in the presence of polybrene (0.8 μg/ml, Sigma, St Louis, MO, USA) (Lian et al., 2010). iPSCs were generated from overnight lentiviral transduction process followed by replacement with human ESC culture medium and then transferred to inactivate mouse embryonic fibroblast (MEF) the day after. After the screening the ESC as iPSC-colonies on the day 20 post-transduction, iPSCs were differentiates into MSC based on a stepwise protocol reported earlier (Lian et al., 2007). Briefly, iPSCs were grown on a gelatinized 10cm dish containing knockout Dulbecco Modified Eagle's Medium (DMEM, GIBCO, Carlsbad, CA, USA) supplemented with 10% serum replacement medium (GIBCO), 10 ng/mL basic fibroblast growth factor (bFGF, GIBCO), 10 ng/mL platelet-derived growth factor AB (PDGF-AB, Peprotech, Rocky Hill, NH, USA), and 10 ng/mL epidermal growth factor (EGF, Peprotech) for 7 days for differentiation. Then the differentiated iPSCs were incubated with CD24-phycoerythrin (PE) and CD105-FITC (BD PharMingen, San Diego, CA, USA) and cells were sorted for $CD24^{-}CD105^{+}$ cells by a fluorescence-activated cell sorting (FACS) system. The $CD24^{-}CD105^{+}$ cells were seeded in 6-well plate under knockout DMEM with 10% fetal calf serum (FCS), bFGF (5 ng/mL), PDGF-AB (10 ng/mL) and EGF (10 ng/mL). Until the cultures were confluent, cells were split and selected for single cell with pLL3.7–green fluorescence protein–positive (GFP^{+}) labelling in 96-well plate. When the clones from a selected cell reached 70% confluence, the cells were reseeded serially to larger cell culture plates or flasks to get sufficient cells. When the cells reached confluent in 175-cm^2 flask, they were regarded as passage 1 and frozen down as cell stocks for further experiments.

Characterized adult human BM-MSCs were purchased commercially (Cat# PT-2501, Cambrex Bioscience, Rockland, ME, USA), which were also provided from Professor Tse's group. BM-MSCs were also cultured with DMEM plus 10% FCS, bFGF (5ng/mL), and EGF (10ng/mL).

Immunohistochemistry

To detect the retention of human iPSC-MSCs and BM-MSCs in rat myocardium, dual immunofluorescent staining was performed on heart sections with mouse anti-HNA monoclonal antibody and cardiac troponin antibody using standard immunohistochemical protocols.

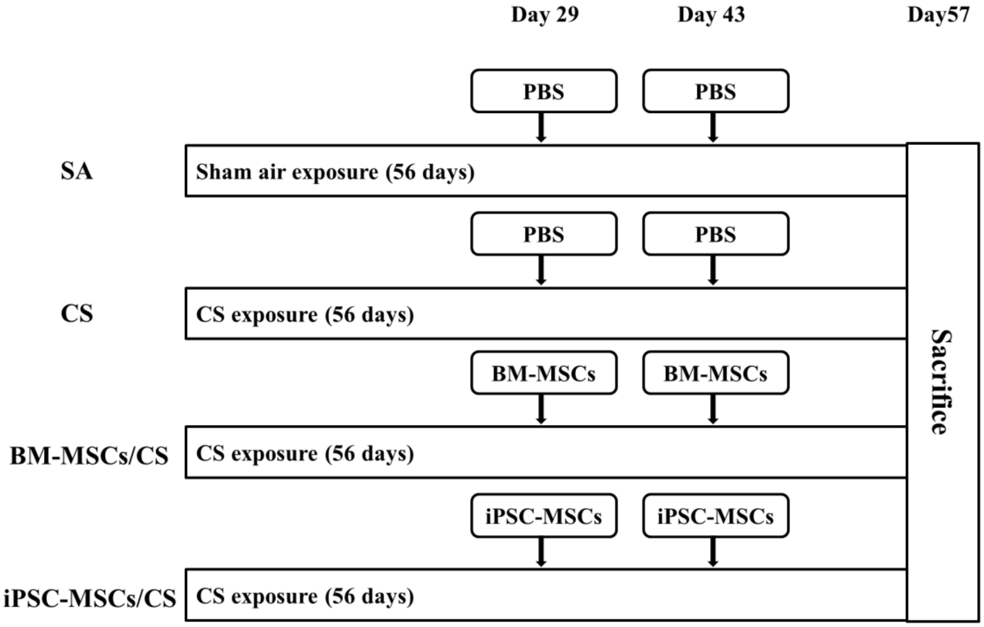

Figure S1 Experimental design for the *in vivo* study.

Schematic diagram of the animal groups showing the CS exposure and MSCs treatment. Male Sprague-Dawley rats were divided into four groups: sham air group (SA), cigarette smoke group (CS), BM-MSC treatment plus CS (BM-MSCs/CS) group and iPSC-MSC treatment plus CS (iPSC-MSCs/CS) group respectively. Rats in the last three groups were to 4% CS for one hour daily for 56 days. 3×10^6 human BM-MSCs or iPSC-MSCs were administrated intravenously at day 29 and day 43. At day 57, echocardiography was conducted before sacrifice.

SUPPLEMENTAL RESULT(S)

The Localization of iPSC-MSCs and BM-MSCs in Heart

Using anti-human nuclear antigen (HNA) antibody, we observed the existence of the human iPSC-MSCs and BM-MSCs in heart sections, showing a greater retention for iPSC-MSCs in the iPSC-MSCs/CS group than for BM-MSCs in the BM-MSCs/CS group at 14 days after tail vein injection (Figure S2).

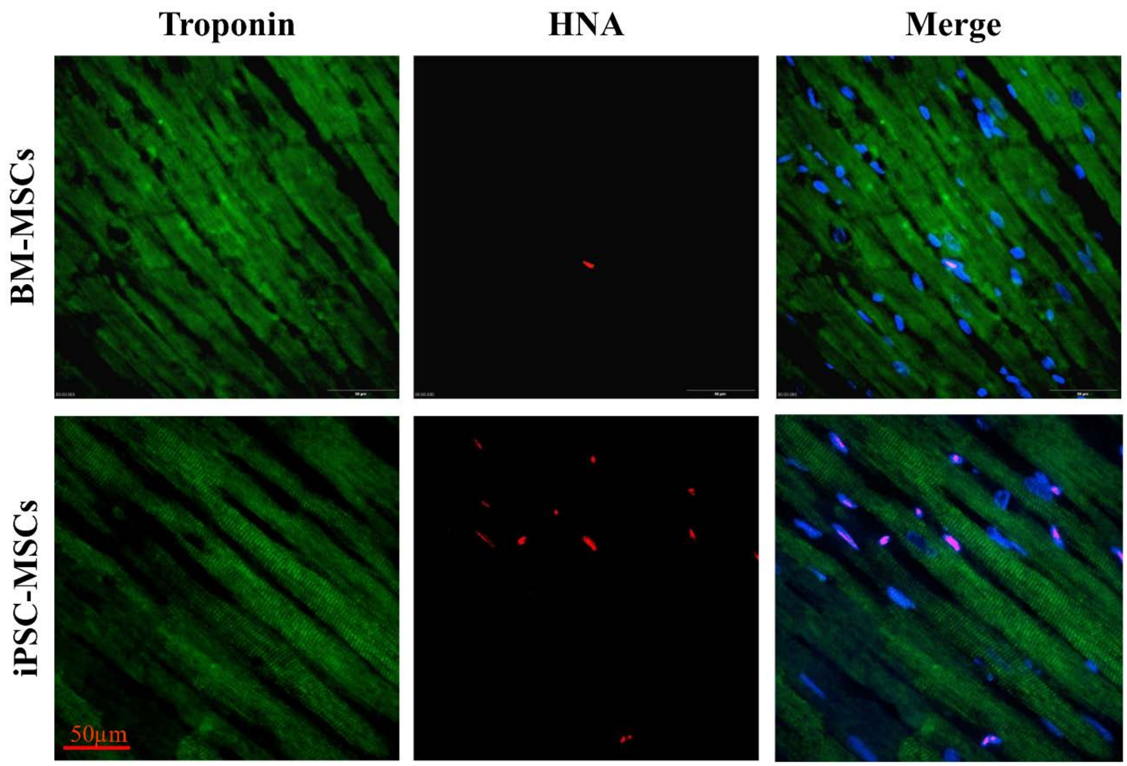

Figure S2. Retention of human BM-MSCs and iPSC-MSCs in myocardium. Top panels, BM-MSCs and bottom panels, iPSC-MSCs were detected by dual fluorescent immunostaining of HNA (red) and cardiac troponin (green) in heart sections at 200× magnifications. The merged images of HNA and troponin markers with DAPI nuclear staining (blue) were shown. Scale bar = 50 μm. HNA, human nuclear antigen.

References:

Lian, Q., Lye, E., Suan Yeo, K., Khia Way Tan, E., Salto-Tellez, M., Liu, T.M., et al. (2007). Derivation of clinically compliant MSCs from CD105+, CD24- differentiated human ESCs. *Stem Cells* 25(2)**,** 425-436. doi: 10.1634/stemcells.2006-0420.

Lian, Q., Zhang, Y., Zhang, J., Zhang, H.K., Wu, X., Zhang, Y., et al. (2010). Functional mesenchymal stem cells derived from human induced pluripotent stem cells attenuate limb ischemia in mice. *Circulation* 121(9)**,** 1113-1123. doi: 10.1161/CIRCULATIONAHA.109.898312.
